# Empowering public health: building advanced molecular surveillance in resource-limited settings through collaboration and capacity-building

**DOI:** 10.3389/frhs.2024.1289394

**Published:** 2024-06-18

**Authors:** Hornel Koudokpon, Boris Lègba, Kevin Sintondji, Islamiath Kissira, Arielle Kounou, Ibrehima Guindo, Kléma Marcel Koné, Mahamadou Abdou, Amadou Koné, Claire Sambou, Honoré Bankolé, Anges Yadouleton, Victorien Dougnon

**Affiliations:** ^1^ Research Unit in Applied Microbiology and Pharmacology of Natural Substances, Research Laboratory in Applied Biology, Polytechnic School of Abomey-Calavi, University of Abomey-Calavi, Cotonou, Benin; ^2^ National Institute of Public Health, Laboratory and Biomedical Research Department, Bamako, Mali; ^3^ University Clinical Research Center, University of Sciences, Techniques and Technology, Bamako, Mali; ^4^ Project Responses to the various Crises Caused by COVID-19 in Mali (RC3-Mali), Health Department, Expertise France, Bamako, Mali; ^5^ Hemorrhagic and Viral Fevers Laboratory, Ministry of Health, Cotonou, Benin

**Keywords:** advanced molecular surveillance system, policy, capacity building, tutoring, Mali

## Abstract

The rapid detection and continuous surveillance of infectious diseases are important components of an effective public health response. However, establishing advanced molecular surveillance systems, crucial for monitoring and mitigating pandemics, poses significant challenges in resource-limited developing countries. In a collaborative effort, research institutions from Benin joined forces with Mali's National Institute of Public Health to implement a state-of-the-art molecular surveillance system in Mali. This approach was characterized by collaboration, multidisciplinarity, and tutoring. Key activities included a comprehensive assessment of infrastructure and human resources through document reviews, interviews, and laboratory visits; the development and validation of Standard Operating Procedures (SOPs) for advanced molecular surveillance following an inclusive approach; capacity-building initiatives for 25 biologists in Mali on sequencing techniques; and international tutoring sessions for eight Malian professionals held in Benin. These collective efforts enabled Mali to establish an advanced molecular surveillance system aligned with the WHO’s global strategy for genomic surveillance. This manuscript aims to share experiences, insights, and outcomes from this initiative, with the hope of contributing to the broader discussion on strengthening global health security through collaborative approaches and capacity-building efforts, particularly in developing countries.

## 1 Introduction

The global public health landscape is continually shaped by the emergence and re-emergence of infectious diseases. Coronavirus Disease 2019 (COVID-19) has put the global public health system to the test, highlighting inadequate preparedness to deal with deadly pandemics. Effective and timely surveillance, particularly in resource-limited settings, is essential for rapid responses to epidemics (1). In this context, molecular surveillance has become an indispensable tool for monitoring and understanding the dynamics of infectious diseases. It provides essential information on the evolution of pathogens, transmission patterns and resistance mechanisms (2, 3).

For instance, employing next-generation sequencing (NGS) to acquire entire genomes serves as a crucial epidemiological technique, allowing the tracking of strain migration, identification of transmission networks, detection of epidemics, and monitoring of diagnostic inconsistencies (4, 5). Advanced molecular surveillance tools were crucial in monitoring the major molecular changes in severe acute respiratory syndrome coronavirus 2 (SARS-CoV-2) during the COVID-19 pandemic (6). COVID-19 disease is the first to apply near real-time whole genome sequencing, with more than 2 million sequences of the entire genome of SARS-CoV-2 generated and shared via the GISAID platform (7, 8).

The World Heath Organisation has recommended the introduction of genomic surveillance of pathogens in every country in the world through its Global Strategy for Genomic Surveillance of Pathogens with Pandemic and Epidemic Potential, 2022–2032. According to this strategy, genomic surveillance is transforming public health action by providing a better understanding of pathogens, how they evolve and how they circulate (9). While many developed nations have exploited advanced molecular surveillance to inform public health interventions (2), the implementation of such systems in developing countries has been hampered by a variety of challenges, including limited infrastructure, inadequate human resources, and limited access to state-of-the-art technologies (10, 11).

Mali, like many other countries in sub-Saharan Africa, faces many infectious disease threats, including emerging pathogens such as SARS-CoV-2 (12). At present, the medical biology laboratory system in Mali plays a crucial role in the response to the pandemic, but it presents certain challenges and limitations that need to be overcome to achieve the objectives of the national response plan and deal with possible future infectious threats. Firstly, although rapid antigen tests, sequencing kits and PCR are used, there is no standardised protocol for advanced molecular surveillance in Mali. This means that there can be variations in the diagnostic and surveillance methods used, which can lead to inconsistencies and gaps in the collection of epidemiological data and the detection of variants (13).

To meet all these challenges, a coordinated approach, and an improvement in the skills of laboratory staff involved in the molecular diagnosis of COVID-19 are required. Aware of the critical importance of advanced molecular surveillance, Mali sought to establish a collaboration with research institutions in Benin, a country with expertise in this field. This collaboration aimed to bridge the gap between the possibilities offered by advanced molecular surveillance and its practical implementation in a resource-limited setting. The partnership was based on the principles of collaboration, multidisciplinarity and tutoring, with the aim of transferring knowledge and skills to strengthen Mali’s capacity in this crucial area. The strategy used was inspired by the World Health Organisation (WHO) considerations for developing a national genomic surveillance strategy or action plan for pathogens with pandemic and epidemic potential (14).

## 2 Methods

The WHO stepwise approach to developing a national genomic surveillance strategy for pathogens with pandemic and epidemic potential involves several key stages: strengthening coordination, conducting a situation analysis, setting goals and objectives, defining the approach to implementation, establishing a monitoring and evaluation framework, developing a costed implementation plan, and mobilizing necessary resources (14). To follow this approach, our methodology incorporated various activities to assess the existing infrastructure, enhance capacity, and ensure the successful implementation of the surveillance system.

### 2.1 Infrastructure and human resources evaluation for the advance molecular surveillance system

The assessment of infrastructure and human resources was designed to offer an exhaustive insight into Mali's existing molecular surveillance infrastructure, outlining strengths and highlighting potential gaps. Furthermore, it aimed to pinpoint key individuals possessing the essential expertise required for advanced molecular surveillance.

#### 2.1.1 Literature review, interviews, and observations

Literature review, interviews and observations was done as described in this study (15). Document collection involved sourcing pertinent reports from government and organizational sources, both online and within the archives of Mali's National Institute of Public Health (INSP). These documents provided a foundational understanding of personnel, infrastructure, and targeted pathogens, forming the basis for a needs assessment. Key informants were strategically chosen from various sectors, including public health organizations, specialized research institutes, universities, and microbiology/virology departments. Notable institutions included the Charles Mérieux Infectiology Center (CICM), the Malaria Research and Training Center (MRTC), the University Clinical Research Center (UCRC), and the Applied Molecular Biology Laboratory (LBMA), selected for their expertise in molecular surveillance and infectious disease diagnostics. Interviews were conducted in two phases: initial online meetings with Mali-based participants and subsequent face-to-face interviews, particularly with institutional heads, to gather detailed information on equipment status, facility quality, and operational realities. This comprehensive methodology facilitated the collection of diverse and in-depth data for the study.

### 2.2 Validation of standardized operating procedures (SOPs)

Standardized Operating Procedures (SOPs) for the molecular surveillance of emerging infections such as COVID-19 was drafted in this step. This plan has been drawn up on the basis of WHO recommendations for setting up a genomic surveillance system (9, 14). This plan is crucial for acquiring vital information to comprehend and manage the disease, identify viral variants, and adapt public health measures accordingly. The proposed plan encompasses the examination of biological properties of variants, including their infectivity, transmissibility, severity, and resistance to treatments. It also entails the collection and analysis of epidemiological data to gain a deeper understanding of virus spread.

### 2.3 Enhancing competencies of 25 biologists in molecular surveillance of pathogens

Building capacity in genomics and bioinformatics is one of the WHO's recommendations for setting up an advanced molecular surveillance system for pathogens with pandemic and epidemic potential (9).

#### 2.3.1 Training approach

The methodology employed for capacity building of 25 biologists in Mali encompassed several stages. The participants were selected based on their profile to ensure equitable sharing of experience and knowledge (16). Among them were microbiologists, medical biology technicians, veterinarians, pharmacists, physicians, epidemiologists, and researchers. The training program itself last two weeks adopted a multifaceted approach, incorporating didactic lectures, hands-on exercises, group work, laboratory visits, discussions, and case analyses. The training program addressed three critical phases of sequencing: pre-analytical, analytical, and post-analytical. The objectives were to provide participants with comprehensive knowledge and practical skills in conducting molecular surveillance activities, including sample collection and preparation, data analysis using bioinformatics tools, and interpretation of results. The success of the training was assessed based on participants’ proficiency in pre-analytical procedures, competence in analytical techniques, accuracy of data interpretation, problem-solving skills, effective communication of results, application of learned skills, and completion of post-training assignments. The selection of topics and content was informed by a thorough needs assessment, aligning training content with the specific needs and gaps identified in the initial stages.

### 2.4 Cross-border tutoring in Benin

The tutoring program, which involved eight Malian biologists, followed a structured methodology. It started with a careful selection of participants who demonstrated their ability to contribute effectively to advanced molecular surveillance activities in Mali. The tutoring approach was designed to be cross-border, involving experts from collaborating institutions in Benin who had valuable experience in disease surveillance. This approach enabled an exchange of experiences in both directions, highlighting the commonalities and shared challenges faced by the two countries during outbreaks. The cross-border tutoring exposed the Malian biologists to established practices of infectious disease surveillance, fostering a learning and knowledge-sharing environment.

## 3 Results

### 3.1 Assessment of infrastructures

A total of four centers were identified as possessing the necessary infrastructure to facilitate advanced molecular surveillance in Mali. These centers include UCRC at the Faculty of Medicine and Dentistry (P3), LBMA at the Faculty of Technical Sciences (P2), the laboratory of the INSP (P2 and mobile P3), and the CICM at base B (P3 and mobile P3). The extent of their surveillance and technical capabilities concerning the targeted pathogens is succinctly summarized in Table 1, 2. Regarding advanced molecular surveillance in Mali, INSP plays a crucial role in actively monitoring SARS-CoV-2 through whole-genome sequencing, antiretroviral and antibiotic resistance analysis, genotyping, healthcare personnel training and awareness, epidemiological surveillance, the provision of diagnostic testing centers, and strengthening molecular diagnostics capacities in all identified centers. The infrastructure evaluation encompassed a comprehensive analysis of laboratory facilities and equipment, evaluating their alignment with international standards. The evaluation revealed a mix of strengths and areas necessitating improvement. Notably, certain laboratories were well-equipped with modern machinery capable of efficiently conducting molecular analyses. However, several laboratories faced challenges related to equipment maintenance and securing a consistent supply of reagents. Furthermore, there was an evident need for biosecurity and biosafety enhancements, underscoring the importance of safe biological material handling and storage.

**Table 1:** Reference laboratories and their infrastructure for molecular surveillance.

Laboratories	Infrastructure
National Institute of Public Health (INSP)	PCR/RT-PCR machines, MinION sequencer, NextSeq Illumina sequencer, Sanger sequencer, Nanodrop spectrophotometer, MALDI-TOF instruments, automated extractor, and flow cytometry devices
University Clinical Research Center (UCRC)	PCR/RT-PCR machines, MinION sequencer, NextSeq Illumina sequencer, Sanger sequencer, Nanodrop spectrophotometer and automated extractor
Malaria Research and Training Center (MRTC)	PCR/RT-PCR machines, MiSeq sequencer, Sanger sequencer, MinION sequencer, Single cell sequencer, Nanodrop spectrophotometer and MALDI-TOF instrument, bioinformatic server
Charles Mérieux Infectiology Center (CICM)	PCR/RT-PCR machines
Laboratory of Applied Molecular Biology (LBMA)	PCR/RT-PCR machines
Central Veterinary Laboratory (LCV)	PCR/RT-PCR machines, Sanger sequencer, NextSeq Illumina sequencer and bioinformatic server

**Table 2:** Reference laboratories for pathogens of interest.

Pathogens	Laboratories	Type of surveillance	Level of surveillance
Cholera	INSP	Active	Molecular surveillance
HIV (human immunodeficieny virus)	INSP, UCRC	Active	Molecular surveillance
Tuberculosis	INSP, CICM, UCRC	Active	Staining/Culture/Molecular surveillance
Plague	LCV	Passive	Immunological, Molecular surveillance
Rift valley fever	UCRC, CICM, LCV, LBMA	Passive	Immunological, Molecular surveillance
Dengue	UCRC, CICM, LBMA, MRTC, INSP	Passive	Molecular surveillance
Avian influenza (bird flu)	LCV	Passive	Molecular surveillance
Avian-origin influenza (H5N1)	LCV	Passive	Molecular surveillance
Pandemic influenza (H1N1)	UCRC, LCV	Passive	Molecular surveillance
Measles	INSP	Passive	Serological
Meningitis	INSP	Passive	Molecular surveillance
Rabies	LCV, UCRC, LBMA	Passive	Immunological, Molecular
Brucellosis	INSP, LCV, CICM	Passive	Molecular surveillance
Anthrax	INSP, LCV	Passive	Molecular surveillance
Chikungunya	UCRC, LBMA, CICM	Passive	Immunological, Molecular
Shigellosis (Bacillary Dysentery)	INSP	Passive	Molecular surveillance
Typhoid fever	INSP	Passive	Molecular surveillance
Leptospirosis	UCRC	Passive	Molecular surveillance
O'nyong-nyong	LBMA	Passive	Immunological,
Malaria	INSP, MRTC, LBMA	Active	Microscopy, Molecular
Yellow fever	INSP, UCRC	Passive	Immunological, Molecular
Lassa fever	LBMA, UCRC, MRTC, CICM, INSP	Passive	Molecular surveillance
Crimean-Congo hemorrhagic fever	UCRC, CICM, INSP, LBMA	Passive	Molecular surveillance
Ebola virus disease	UCRC, CICM	Passive	Molecular surveillance
Marburg virus disease	UCRC, CICM, INSP	Passive	Molecular surveillance
COVID-19	INSP, UCRC, CICM, LBMA	Active	Molecular surveillance
Zika virus	UCRC, CICM, LBMA, INSP	Passive	Molecular surveillance
West Nile virus disease	UCRC, CICM	Passive	Molecular surveillance

### 3.2 Assessment of resource persons

The evaluation of human resources aimed to identify individuals possessing the requisite expertise for effective molecular surveillance. Through interviews and discussions with key stakeholders, highly skilled personnel were identified, many of whom had received prior training in molecular biology and epidemiology. However, the assessment also revealed that a limited number of staff members possess proficient skills in molecular surveillance techniques. For instance, at the INSP Medical Biology Laboratory, there are approximately 66 members, but only three are formally involved in Molecular Surveillance. All employees in various INSP laboratories possess general experience in molecular diagnostic tests such as PCR. However, few employees have specific expertise in bioinformatics.

This evaluation not only sheds light on the strengths and weaknesses of Mali’s molecular surveillance infrastructure but also underscores the importance of continuous training and capacity-building to ensure the effective implementation of advanced molecular surveillance practices. Furthermore, it highlights the need for strategic resource allocation to enhance biosecurity and biosafety measures within these laboratories, thereby safeguarding against potential risks associated with handling biological materials. Following the assessment, key recommendations: (1) the establishment and implementation of a formal collaboration framework between INSP and partner laboratories, (2) the reorganization of roles and responsibilities of personnel at INSP, (3) the regular supply of consumables and reagents to partner laboratories for molecular analyses, (4) the development of continuous training programs to enhance technical skills in molecular surveillance, (5) the standardization of molecular surveillance protocols and data sharing, (6) the formation of an implementation and monitoring committee for surveillance activities, and (7) the establishment of a data sharing mechanism for consideration in policy decisions.

### 3.3 Advanced molecular surveillance system design for Mali, featuring standard operating procedures

The plan delineates a comprehensive approach to molecular surveillance for COVID-19 and other pathogens of interest (17). It provides detailed descriptions of the roles and responsibilities of key stakeholders, including the national coordination structure, the national reference laboratory, and surveillance sites. The surveillance strategy is outlined meticulously, encompassing clinical surveillance methodology, selection of sentinel sites, specimen collection, and sampling standards. The molecular identification of pathogens, including COVID-19, is thoroughly addressed, covering sentinel laboratories, sample sequencing, and isolate preservation. Quality assurance is also discussed throughout the pre-analytical, analytical, and post-analytical phases. The collection and management of epidemiological data, along with reporting and notifications, are detailed in the data management section. Monitoring and evaluation of the surveillance system are addressed, with an emphasis on timely reporting, sample management, and quality controls.

To support the plan, Standard Operating Procedures (SOPs) have been drafted using bibliographic research to study existing monitoring programmes. These SOPs encompass the pre-analytical, analytical, and post-analytical phases. The procedures cover epidemiological surveillance, laboratory techniques and biosafety procedures. Each procedure is summarized in five key points: context, objectives, roles and responsibilities, equipment lists and step-by-step execution. The procedures developed will play a central role in the molecular surveillance of pathogens in Mali by ensuring the consistency, quality, traceability, compliance, and continuity of surveillance activities.

The drafts of the SOPs drawn up were validated at a two-day validation workshop held at Mali's INSP, with resource persons identified in the 4 central laboratories that could be involved in advanced molecular surveillance. The SOPs were reviewed and validated with input from members of each involved laboratory, taking into account their resources. Each SOP has been experimentally validated by at least one laboratory. As a result, they will help to strengthen national capacities for pathogen detection and surveillance, which will facilitate informed decision-making on disease prevention and control.

### 3.4 Training outcomes

Participants gained a comprehensive understanding of Polymerase Chain Reaction (PCR) techniques tailored for molecular surveillance, cutting-edge sequencing methodologies, with a particular emphasis on Next-Generation Sequencing (NGS) applications. This knowledge is paramount for advanced molecular surveillance practices. Participants received in-depth training on the preparatory stages preceding sequencing, on the analytical aspects of sequencing, particularly emphasizing NGS techniques. Participants developed the ability to navigate complex sequencing data effectively and on harnessing online resources for in-depth viral genome analysis. In an era of rapidly evolving data, this skill is indispensable. Quality control is paramount in molecular surveillance. Participants learned the intricacies of maintaining high-quality sequence data and implementing rigorous quality control measures. Quality control measures, including the implementation of internal controls, regular instrument maintenance, and rigorous bioinformatics QC checks, were emphasized to ensure the reliability and accuracy of sequencing data.

### 3.5 Training assessment

The training's effectiveness was evaluated through pre-tests and post-tests assessing participants’ knowledge in the pre-analytical, analytical, and post-analytical phases of sequencing. The assessment yielded highly positive results. The results showed an improvement in the number of participants achieving a passing grade or score after training, with 17 participants meeting the criteria compared to 2 before training (Table 3).

**Table 3:** Pre-test and post-test results.

Assessment criteria	Before training	After training
Passing grade/Score achieved	2 (8%)	17 (68%)

Participants expressed a high level of satisfaction with the training, underscoring its success in meeting their expectations. This positive feedback reflects the quality of the instructors and the effectiveness of the pedagogical approach. The limited program duration (two weeks) posed constraints, particularly due to the varying levels of prior knowledge among participants. To optimize future training programs, participants recommended selecting remote training locations to maximize concentration, organizing visits to neighboring countries with well-established surveillance programs, involving all laboratory technicians in ongoing training, allocating time for practical laboratory work, and reinforcing quality control elements of the training curriculum.

### 3.6 Impact of the cross-border tutoring

During their cross-border tutoring in Benin, participants explored URMAPha and the Viral Hemorrhagic Fevers Laboratory, gaining insights into advanced molecular surveillance. Experiences shared served as inspiration for fortifying Mali's surveillance system, revealing similarities in healthcare systems and challenges faced during epidemics. Discussions highlighted international collaboration’s importance for quality control and data management in serological and molecular testing. The visit to Mycobacteria Reference Laboratory (LRM) deepened understanding of tuberculosis surveillance, while hands-on activities enhanced practical skills for surveillance of various pathogens. These exchanges fostered collaborations, enriching knowledge and strengthening healthcare systems in both countries.

## 4 Discussions

The establishment of an advanced molecular surveillance system addresses a critical need for global health: enhancing Mali's healthcare system's capacity for screening, detecting variants, conducting advanced molecular surveillance, and developing national documents (strategic and operational) for advanced molecular surveillance. This necessity aligns with the recommendations of the technical Guide for Integrated Disease Surveillance and Response in Mali (13) and is consistent with WHO recommendations for implementing a genomic pathogen surveillance system, particularly in the aftermath of the COVID-19 pandemic (9).

In the implementation of this advanced molecular surveillance system, the expertise of reference institutions and laboratories in Benin has been sought, as this country has recently made significant progress in the establishment of molecular surveillance systems. This progress is particularly notable through the Benin Reference Laboratory for Viral Hemorrhagic Fevers and the Research Unit in Applied Microbiology and Natural Substance Pharmacology Unit. Benin has generated extensive data concerning disease progression, SARS-CoV-2 variants, and the country's diagnostic capabilities. These insights have enabled the identification of trends, risk factors, and concerning variants, all of which have contributed significantly to the implementation of effective public health measures. Additionally, there have been substantial improvements in diagnostic infrastructure and screening protocols, ensuring accurate monitoring of the epidemic (18–21).

To support the implementation of advanced molecular systems at country level, the WHO has developed a step-by-step guide that outlines key considerations and proposes an approach for countries to develop a national genomic surveillance strategy or action plan for pathogens with pandemic and epidemic potential. These include ensure strong national leadership, financial commitment and governance framework, Focus on public health decision-making, target all relevant pathogens with priority pathogen use cases, strengthen data management, and promote data sharing and collaboration (14). These considerations highlight the need to take stock of the situation before setting up a monitoring system. The evaluation of existing infrastructure and human resources within Mali's public healthcare system has provided invaluable insights. While some laboratories have demonstrated their capability for advanced molecular surveillance, challenges related to equipment maintenance, reagent supply, and biosafety protocols require special attention. These findings also underscore the significance of not only having state-of-the-art equipment but also maintaining them effectively. Furthermore, there is a notable limitation in the expertise of human resources in advanced molecular surveillance, particularly in sequencing and bioinformatics techniques. Similarly, infrastructure limitations and lack of trained personnel were identified as major challenges for laboratory establishment in Nigeria (22). Actions must be initiated to address the identified gaps and strengthen Mali’s advanced molecular system. These actions should involve capacity-building for key stakeholders involved in advanced molecular surveillance. Strategic Objective 2 of the Global Strategy for Genomic Surveillance of Potential Pandemic and Epidemic Pathogens aims to strengthen the workforce to ensure speed, scalability, and service quality. To achieve this objective, the WHO recommends deploying training modules in genomics and bioinformatics and promoting communities of practice and knowledge exchange (9). These two recommendations formed the basis for capacity-building activities. Trained personnel play a crucial role in ensuring the sustainability of any laboratory (22). The capacity-building program conducted for these 25 biologists represents a significant achievement, as we observed improvement in participants’ knowledge on the pre-analytical, analytical, and post-analytical phases of sequencing. Similar training content was used in Pakistan to build up a genomic surveillance platform for SARS-CoV-2 (23). This strengthened expertise can serve as a foundation for more effective surveillance and response to infectious diseases. However, it is crucial to acknowledge that continuous training and professional development will be necessary to keep pace with the evolving landscape of molecular surveillance technologies.

Collaboration with local partners and international orga-nisations is instrumental in sustaining a laboratory inresource-limited settings (22). The cross-border tutoring program conducted in Benin provided invaluable exposure to established practices in infectious disease surveillance. It facilitated a bidirectional exchange of experiences, highlighting the similarities and common challenges faced by both countries, especially during epidemic episodes. These observations are in line with the key characteristics for a resilient public health systems capable of preparing for, detecting, managing, and recovering from emergencies such as COVID-19 (24).This experience underscored the potential of regional collaborations to enhance surveillance and response capabilities, especially as the WHO recommends strengthening links between networks at local, regional and global level to maximise linkage for timely added value in the wider surveillance architecture (14).

### 4.1 Policy recommendations

#### 4.1.1 Monitoring and continuous improvement of the surveillance system

To ensure the continuous enhancement of surveillance quality, a structured strategy should be developed to govern the creation of technical annexes, as well as mechanisms to monitor and evaluate the performance of the federated system. Formative and summative assessments of the federated system should be conducted to guide decision-making processes effectively. To evaluate the effectiveness of the advanced molecular surveillance program and measure the achievement of set objectives, the implementation of a robust monitoring and evaluation system is crucial. Relevant performance indicators should be defined to measure the impact of activities and adjust strategies based on outcomes. To ensure the effectiveness of the advanced molecular surveillance program, it’s crucial to monitor various key performance indicators, including compliance with established technical annexes, frequency of monitoring and evaluation assessments, alignment of actions with assessment findings, achievement rate of set objectives, data quality of surveillance, response time to emerging threats, impact of capacity building efforts, efficiency of resource utilization, and stakeholder satisfaction.

#### 4.1.2 Ongoing capacity building for trained biologists

To keep the trained biologists up to date with the latest advancements in molecular surveillance, a continuous training plan should be implemented. This plan should include regular workshops, seminars, and specialized training sessions covering emerging technologies and surveillance methods. These training opportunities will help biologists stay informed about scientific and technological developments, enhancing their skills in pathogen detection, sequencing, and analysis. To broaden the representation of central laboratories and gain a more comprehensive view of the epidemiological landscape, it is advisable to increase the number of biologists involved in the advanced molecular surveillance program. The inclusion of additional experts will diversify expertise, enhance response capacity, and improve data collection and analysis.

#### 4.1.3 National and international partnerships: a crucial element in a sustainable advanced molecular surveillance strategy in developing countries

It is recommended to maintain and strengthen the partnerships between institutions involved in the advanced molecular surveillance in Mali. Effective coordination and seamless communication between central laboratories, INSP, and other key partners, including academic institutions, will maximize the program’s impact and ensure a holistic and integrated approach to pathogen surveillance. However, the importance of continuous sharing of information, experiences, and expertise to enhance advanced molecular surveillance practices in the region was highlighted. A comprehensive collaborative approach is essential for addressing emerging epidemics and strengthening national and regional healthcare systems (14). Collaboration between Beninese and Malian healthcare experts in the field could pave the way for broader partnerships aimed at strengthening healthcare systems and better preparing both countries to address future health crises.

## 5 Conclusion

The effective establishment of advanced molecular surveillance in Mali, with valuable support and insights from Benin, underscores the tremendous potential of international collaboration and tutoring in enhancing healthcare infrastructure and expertise, particularly in resource-constrained settings. This accomplishment not only contributes significantly to the global health security dialogue but also emphasizes the critical need to align surveillance systems, invest in capacity development, and cultivate partnerships to adeptly respond to emerging health risks.

## Data Availability

The raw data supporting the conclusions of this article will be made available by the authors, without undue reservation.

## References

[1] Berg MG Olivo A Forberg K Harris BJ Yamaguchi J Shirazi R Advanced molecular surveillance approaches for characterization of blood borne hepatitis viruses. PLoS One. (2020) 15(7):e0236046. 10.1371/journal.pone.0236046 32678844 PMC7367454

[2] Gwinn M MacCannell DR Khabbaz RF. Integrating advanced molecular technologies into public health. J Clin Microbiol. (2017) 55(3):703–14. 10.1128/JCM.01967-16 28031438 PMC5328438

[3] Jackson BR Tarr C Strain E Jackson KA Conrad A Carleton H Implementation of nationwide real-time whole-genome sequencing to enhance listeriosis outbreak detection and investigation. Clin Infect Dis. (2016) 63(3):380–6. 10.1093/cid/ciw242 27090985 PMC4946012

[4] Casto AM Adler AL Makhsous N Crawford K Qin X Kuypers JM Prospective, real-time metagenomic sequencing during norovirus outbreak reveals discrete transmission clusters. Clin Infect Dis. (2019) 69(6):941–8. 10.1093/cid/ciy1020 30576430 PMC6735836

[5] Thézé J Li T du Plessis L Bouquet J Kraemer MUG Somasekar S Genomic epidemiology reconstructs the Introduction and spread of Zika virus in Central America and Mexico. Cell Host Microbe. (2018) 23(6):855–864.e7. 10.1016/j.chom.2018.04.017 29805095 PMC6006413

[6] Lina B. Les différentes phases de l’évolution moléculaire et antigénique des virus SARS-CoV-2 au cours des 20 mois suivant son émergence. Bulletin de L’Académie Nationale de Médecine. (2022) 206(1):87–99. 10.1016/j.banm.2021.11.002 PMC8629187 34866635

[7] Flores-Alanis A Cruz-Rangel A Rodríguez-Gómez F González J Torres-Guerrero CA Delgado G Molecular epidemiology surveillance of SARS-CoV-2: mutations and genetic diversity one year after emerging. Pathogens. (2021) 10(2):184. 10.3390/pathogens10020184 33572190 PMC7915391

[8] Africa CDC. Interim Operational Guidance on SARS-CoV-2 Genomic Surveillance in Africa: An Updated Guide . Africa CDC and WHO/AFRO (2021). Available online at: https://africacdc.org/download/interim-operational-guidance-on-sars-cov-2-genomic-surveillance-in-africa-an-updated-guide/ (cited September 4, 2023)

[9] WHO. Global Genomic Surveillance Strategy for Pathogens with Pandemic and Epidemic Potential, 2022–2032. Geneva: World Health Organization (2022). Available online at: https://www.who.int/publications-detail-redirect/9789240046979 (cited September 3, 2023) 10.2471/BLT.22.288220 PMC8958828 35386562

[10] Akande OW Carter LL Abubakar A Achilla R Barakat A Gumede N Strengthening pathogen genomic surveillance for health emergencies: insights from the world health organization’s regional initiatives. Front Public Health. (2023) 11:1–8. 10.3389/fpubh.2023.1146730 PMC10289157 37361158

[11] Inzaule SC Tessema SK Kebede Y Ouma AEO Nkengasong JN. Genomic-informed pathogen surveillance in Africa: opportunities and challenges. Lancet Infect Dis. (2021) 21(9):e281–9. 10.1016/S1473-3099(20)30939-7 33587898 PMC7906676

[12] Koné A Diallo D Kané F Diarra B Coulibaly TA Sameroff SC Dynamics of SARS-CoV-2 variants characterized during different COVID-19 waves in Mali. IJID Regions. (2023) 6:24–8. 10.1016/j.ijregi.2022.11.009 36448028 PMC9691504

[13] Ministère de la Santé et du Développement Social du Mali. Guide Technique Pour la Surveillance Integree de la Maladie et la Riposte au mali. Brazzaville, République du Congo: Bureau régional de l'OMS pour l'Afrique Groupe Prévention et lutte contre les maladies (2021). p. 901.

[14] WHO. Considerations for Developing a National Genomic Surveillance Strategy or Action Plan for Pathogens with Pandemic and Epidemic Potential—World | ReliefWeb . Geneva: World Health Organization (2023). Available online at: https://reliefweb.int/report/world/considerations-developing-national-genomic-surveillance-strategy-or-action-plan-pathogens-pandemic-and-epidemic-potential (cited September 3, 2023)

[15] Keating P Pharris A Leitmeyer K Angelis SD Wensing A Amato-Gauci AJ Assessment of HIV molecular surveillance capacity in the European union, 2016. Eurosurveillance. (2017) 22(49):17. 10.2807/1560-7917.ES.2017.22.49.17-00269 29233253 PMC5727594

[16] Sanchez JL Johns MC Burke RL Vest KG Fukuda MM Yoon IK Capacity-building efforts by the AFHSC-GEIS program. BMC Public Health. (2011) 11(2):S4. 10.1186/1471-2458-11-S2-S4 21388564 PMC3092414

[17] OMS WH. Global Genomic Surveillance Strategy for Pathogens with Pandemic and Epidemic Potential 2022-2032: Consultation Meeting Report, 2021. Geneva: World Health Organization (2023). p. 26. 10.2471/BLT.22.288220 PMC8958828 35386562

[18] Yadouleton A Sander AL Moreira-Soto A Tchibozo C Hounkanrin G Badou Y Limited specificity of serologic tests for SARS-CoV-2 antibody detection, Benin. Emerg Infect Dis. (2021) 27(1):233. 10.3201/eid2701.203281 33261717 PMC7774555

[19] Houngbégnon P Nouatin O Yadouléton A Hounkpatin B Fievet N Atindégla E Interest of seroprevalence surveys for the epidemiological surveillance of the SARS-CoV-2 pandemic in African populations: insights from the ARIACOV project in Benin. Trop Med Int Health. (2023) 28:508–16. 10.1111/tmi.13895 37243412

[20] Yadouleton A Sander AL Adewumi P de Oliveira Filho EF Tchibozo C Hounkanrin G Emergence of SARS-CoV-2 Delta variant, Benin, May–July 2021. Emerg Infect Dis. (2022) 28(1):205. 10.3201/eid2801.211909 34807815 PMC8714210

[21] Sander AL Yadouleton A de Oliveira Filho EF Tchibozo C Hounkanrin G Badou Y Mutations associated with SARS-CoV-2 variants of concern, Benin, early 2021. Emerg Infect Dis. (2021) 27(11):2889. 10.3201/eid2711.211353 34463240 PMC8544961

[22] Isah MB Muhammad Z Lawan MM Alkhamis AI Goni BW Oakley SS Setting up a state-of-the-art laboratory in resource limited settings: a case study of the biomedical science research and training centre in northeast Nigeria. Eur J Neurosci. (2023) 59(7):1405–859. 10.1111/ejn.16260 38311832

[23] Khan W Kabir F Kanwar S Aziz F Muneer S Kalam A Building up a genomic surveillance platform for SARS-CoV-2 in the middle of a pandemic: a true north–south collaboration. BMJ Global Health. (2023) 8(11):e012589. 10.1136/bmjgh-2023-012589 37984892 PMC10660681

[24] Doble A Sheridan Z Razavi A Wilson A Okereke E. The role of international support programmes in global health security capacity building: a scoping review. PLOS Global Public Health. (2023) 3(4):e0001763. 10.1371/journal.pgph.0001763 37018186 PMC10075474

